# Receptor-Mediated Host Cell Preference of a Bat-Derived Filovirus, Lloviu Virus

**DOI:** 10.3390/microorganisms8101530

**Published:** 2020-10-05

**Authors:** Yoshihiro Takadate, Rashid Manzoor, Takeshi Saito, Yurie Kida, Junki Maruyama, Tatsunari Kondoh, Hiroko Miyamoto, Hirohito Ogawa, Masahiro Kajihara, Manabu Igarashi, Ayato Takada

**Affiliations:** 1 Division of Global Epidemiology, Research Center for Zoonosis Control, Hokkaido University, Sapporo 001–0020, Japan; ytakadate@czc.hokudai.ac.jp (Y.T.); r_manzoor@hotmail.com (R.M.); t.saito@czc.hokudai.ac.jp (T.S.); yurie-kida@czc.hokudai.ac.jp (Y.K.); jumaruya@UTMB.EDU (J.M.); 1108.mr.tk@gmail.com (T.K.); hirom@czc.hokudai.ac.jp (H.M.); kajihara@czc.hokudai.ac.jp (M.K.); igarashi@czc.hokudai.ac.jp (M.I.); 2 Hokudai Center for Zoonosis Control in Zambia, School of Veterinary Medicine, University of Zambia, P.O. Box 32379, Lusaka 10101, Zambia; hogawa@okayama-u.ac.jp; 3 Department of Disease Control, School of Veterinary Medicine, University of Zambia, P.O. Box 32379, Lusaka 10101, Zambia; 4 Global Station for Zoonosis Control, Global Institution for Collaborative Research and Education, Hokkaido University, Sapporo 001–0020, Japan

**Keywords:** filovirus, lloviu virus, *Miniopterus* sp., bat, insectivorous bat, host range, Niemann–Pick C1, glycoprotein

## Abstract

Lloviu virus (LLOV), a bat-derived filovirus that is phylogenetically distinct from human pathogenic filoviruses such as Ebola virus (EBOV) and Marburg virus (MARV), was discovered in Europe. However, since infectious LLOV has never been isolated, the biological properties of this virus remain poorly understood. We found that vesicular stomatitis virus (VSV) pseudotyped with the glycoprotein (GP) of LLOV (VSV–LLOV) showed higher infectivity in one bat (*Miniopterus* sp.)-derived cell line than in the other bat-derived cell lines tested, which was distinct from the tropism of VSV pseudotyped with EBOV (VSV–EBOV) and MARV GPs. We then focused on the interaction between GP and Niemann–Pick C1 (NPC1) protein, one of the cellular receptors of filoviruses. We introduced the *Miniopterus* bat and human NPC1 genes into NPC1-knockout Vero E6 cells and their susceptibilities to the viruses were compared. The cell line expressing the bat NPC1 showed higher susceptibility to VSV–LLOV than that expressing human NPC1, whereas the opposite preference was seen for VSV–EBOV. Using a site-directed mutagenesis approach, amino acid residues involved in the differential tropism were identified in the NPC1 and GP molecules. Our results suggest that the interaction between GP and NPC1 is an important factor in the tropism of LLOV to a particular bat species.

## 1. Introduction

Ebola virus (EBOV) and Marburg virus (MARV), belonging to the genera *Ebolavirus* and *Marburgvirus*, respectively, of the family *Filoviridae*, cause severe hemorrhagic fever in humans and nonhuman primates [1,2]. While the ecology of EBOV and MARV is largely unknown, some species of fruit bats are suspected to be natural reservoirs of these viruses [3,4,5,6,7]. In 2002, a novel filovirus, Lloviu virus (LLOV), phylogenetically distinct from the viruses in the genera *Ebolavirus* and *Marburgvirus*, was discovered in carcasses of insectivorous bats (Schreiber’s bent-winged bat: *Miniopterus schreibersii*) in Spain [8] and this virus was designated a new filovirus member belonging to the genus *Cuevavirus* [2,8]. In 2016, LLOV was detected again in the same species of bats in Hungary [9]. More recently, the full-length genomes of previously unknown filoviruses (i.e., Bombali and Mengla viruses) were discovered in bats [10,11]. Taken together, the frequent detection of filoviruses in bats suggests that this animal is closely related to the ecology of filoviruses.

Infectious LLOV particles have never been isolated, and the biological properties of LLOV are still unclear. Previous studies suggest that the envelope glycoprotein (GP) of LLOV is the only glycoprotein responsible for virus entry into cells [12,13]. Like other filoviruses, LLOV GP is thought to play a major role in the replication of filoviruses and to have the potential to mediate virus entry into mammalian cells, including those of humans and bats [13,14]. It has also been shown that viral protein (VP) 24 and VP35, which are known to suppress innate immune responses and to play important roles in the pathogenicity of EBOV and/or MARV [15], are also encoded in the LLOV genome. LLOV VP24 and VP35 were shown to antagonize immune responses in human cells [16]. These findings suggest that LLOV has the capacity to infect a wide variety of mammalian cells and may be a potential pathogen for humans.

In general, the host range and tropism of viruses (i.e., cell susceptibility) are determined by multiple viral and host factors. One of the important steps is the entry of viruses into cells, which is generally mediated by interactions between viral surface proteins and host cell receptors [17]. Previous studies have suggested that each filovirus has a different preference for bat species [18,19,20], which may be principally determined by the interactions between GPs and filovirus receptors. Previously, we compared the susceptibilities of bat-derived cell lines to filoviruses, using vesicular stomatitis viruses (VSVs) pseudotyped with GPs, and found that filoviruses might have preferential tropism to some particular bat species depending on the virus species. In this study, we focused on the interaction between LLOV GP and a host cellular receptor, Niemann–Pick C1 (NPC1) protein [21,22], and investigated the molecular mechanisms underlying the cell preference of LLOV. Here, we show that the heterogeneity of domain C of NPC1 (NPC1-C), which interacts with filovirus GP [23,24], is important for the distinctive cell tropism of LLOV to the particular bat cell line.

## 2. Materials and Methods 

### 2.1. Cells

Vero E6 cells were grown in Dulbecco’s modified Eagle medium (Sigma, Saint Louis, MO, USA) supplemented with 10% fetal calf serum (FCS) (Cell Culture Bioscience), 100 U/mL penicillin, and 0.1 mg/mL streptomycin (Gibco). Bat-derived cell lines were established as described previously [25,26]. All of the bat cell lines were grown in Roswell Park Memorial Institute 1640 medium (Sigma) supplemented with 10% FCS, 100 units/mL penicillin, and 0.1 mg/mL streptomycin. All cell lines were cultured at 37 °C in a 5% CO2 incubator. The origins of these cell lines are shown in Table 1. SuBK12-08 cells were identified by morphology, habitat, and BLAST searches using the sequences of the cytochrome *b* gene [27]. Vero E6/NPC1-KO cl.19 and Vero E6/NPC1-KO cl.19, expressing exogenous NPC1 derived from HEK293T or SuBK12-08, were generated and maintained as described previously [20,28]. 

### 2.2. Viruses

Using VSV containing the green fluorescent protein gene instead of the receptor binding VSV G protein gene, pseudotyped viruses with GPs of EBOV (Mayinga), MARV (Angola), and LLOV (Asturias) (VSV–EBOV, –MARV, and –LLOV) were generated as described previously [12,13]. The mutant GP genes were constructed by site-directed mutagenesis with KOD One (TOYOBO). VSVs pseudotyped with filovirus GPs were preincubated with an anti-VSV G monoclonal antibody, VSV-G [N] 1–9 [29], to abolish the background infectivity of parental VSV. Pseudotyped VSVs were inoculated into confluent cell monolayers cultured on 96-well plates, and infectious units (IU) in each cell line were determined 20 h later by counting the number of GFP-expressing cells under a fluorescent microscope. The relative infectivity of pseudotyped VSVs in bat-derived cell lines was determined by setting the GFP-positive cell number of Vero E6 cells infected with each virus to 1.0. The relative infectivities of pseudotyped VSVs in Vero E6/NPC1-KO cl.19 expressing exogenous NPC1 were determined by setting the GFP-positive cell number of wildtype HEK293T NPC1-expressing cells infected with each virus to 1.0.

### 2.3. Bat NPC1 Genes

We used the nucleotide sequences of NPC1 genes derived from SuBK12-08, YubFKT1, BKT1, FBKT1, ZFBK13-76E, ZFBK11-97, ZFBK15-137RA, and DemKT1, which were determined previously (GenBank accession numbers; LC462997, LC462271, LC462998, LC462999, LC462993, LC462994, LC462995, and LC462996, respectively [20]). Total RNA was extracted from SuBK12-08 cells using ISOGEN (Nippongene) and mRNA was reverse transcribed with Superscript IV (Invitrogen). The NPC1 gene of SuBK12-08 was amplified with KOD One and inserted into a pSP72 (Promega, Madison, WI, USA) plasmid vector. After sequence confirmation, domain C of the NPC1 gene fragment derived from SuBK12-08 cells (amino acid residues 377–624 [373–620 in HEK293T NPC1 numbering]) and the NPC1 gene fragment derived from HEK293T cells (amino acid residues 1–372 and 621–1279) were amplified with KOD One using specific primers with the HA tag sequence. Then these NPC1 gene fragments were inserted into the pMXs-puro retroviral vector (Cell Biolabs. San Diego, CA, USA) to construct pMXs-chimeric HEK293T/SuBK12-08 NPC1. An In-Fusion cloning kit (BD Clontech) was used to construct the retroviral vectors carrying NPC1 genes. The plasmids of mutant NPC1 genes were constructed by site-directed mutagenesis with KOD One. After sequence confirmation, these mutant genes were inserted into the retroviral vector.

### 2.4. Generation of Stable Cell Lines Expressing NPC1 Proteins

Vero E6/NPC1-KO cl.19 cells expressing human NPC1, HEK293T/SuBK12-08 NPC1, and their mutants were generated as described previously [20]. Briefly, HEK293T-derived Platinum-GP cells (Cell Biolabs) were cotransfected with pMXs-puro encoding NPC1 genes and the expression of plasmid pCAGGS encoding the VSV G using Lipofectamine 2000 (Invitrogen, Carlsbad, CA, USA). Forty-eight hours later, the culture supernatants containing retroviruses were collected and used to infect Vero E6/NPC1-KO cl.19, an NPC1-knockout cell line [28]. Transduced cells stably expressing exogenous NPC1 were selected with a growth medium containing 10.0 μg/mL puromycin (Sigma-Aldrich). 

### 2.5. Western Blotting

Each virus was subjected to sodium dodecyl sulfate–polyacrylamide gel electrophoresis (SDS-PAGE) followed by Western blotting with a mouse anti-EBOV GP monoclonal antibody (ZGP42/3.7) [30], a mouse anti-LLOV GP monoclonal antibody (LGP14–2) [13], a mouse anti-VSV matrix protein antibody (VSV-M 195–2) [31] and horseradish peroxidase (HRP)-conjugated goat anti-mouse IgG (115–035–062, Jackson ImmunoResearch, PA, USA). For NPC1 and β actin detection, each cell lysate was subjected to SDS-PAGE followed by Western blotting with an HRP-conjugated rat anti-HA monoclonal antibody (3F10, Sigma), a mouse anti-β actin monoclonal antibody (A1978, Sigma), and an HRP-conjugated goat anti-mouse IgG (115–035–062, Jackson ImmunoResearch). The bound antibodies were visualized with Immobilon Western (Millipore, Burlington, MA, USA).

### 2.6. Statistical Analysis

All statistical analyses were performed using R software (Version 3.5.2) [32]. For a comparison of the viral infectivities of pseudotyped VSVs, the Student *t*-test or a one-way analysis of variance followed by Dunnett’s test was used. *p*-values of less than 0.05 were considered to be significant.

## 3. Results

### 3.1. Susceptibility of Bat-Derived Cell Lines to VSV–EBOV, –MARV, and –LLOV

**Figure 1:**
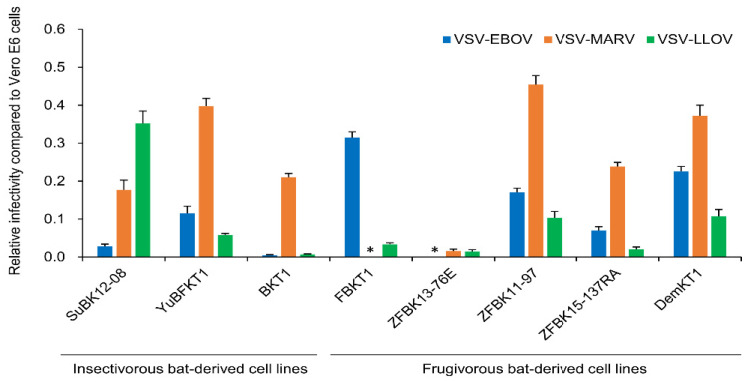
Susceptibilities of bat-derived cell lines to vesicular stomatitis virus (VSV) pseudotyped with filovirus glycoproteins (GPs). Vero E6 and bat-derived cells were infected with VSVs pseudotyped with filovirus GPs (VSV–Ebola virus (EBOV), –Marburg virus (MARV), and –Lloviu virus (LLOV)). The relative infectivity of pseudotyped VSVs in bat-derived cell lines, compared to Vero E6 cells, was determined as described in Section 2. Each experiment was conducted three times, and average and standard deviations are shown. Asterisks represent IUs under the limit of detection (20 IU/mL).

Using replication-incompetent VSVs pseudotyped with GPs of EBOV, MARV, and LLOV, we investigated GP-dependent tropism, which is the primary determinant for cell susceptibility to filoviruses. Vero E6 cells, which are generally used for filovirus studies, and eight bat-derived cell lines of different origins (i.e., three from insectivorous bat species in the suborder Microchiroptera and five from frugivorous bat species in the suborder Megachiroptera) were used to compare their susceptibilities (Table 1 and Figure 1). We found that SuBK12-08, a cell line derived from *Miniopterus* sp., showed higher susceptibility to VSV–LLOV than to VSV–EBOV and –MARV, although cell lines derived from other bat species, including two insectivorous bat species, did not show such preferential susceptibility to VSV–LLOV. Interestingly, VSV–EBOV showed remarkably lower infectivity in SuBK12-08 while VSV–MARV infected most of the bat-derived cells with relatively high infectivity, except for FBKT1 and ZFBK13-76E cells. 

### 3.2. Amino Acid Sequences of Domain C of Bat NPC1 Orthologues and Susceptibilities of Vero E6 Cell Lines Expressing Exogenous NPC1 Proteins to VSV–EBOV and –LLOV

**Figure 2:**
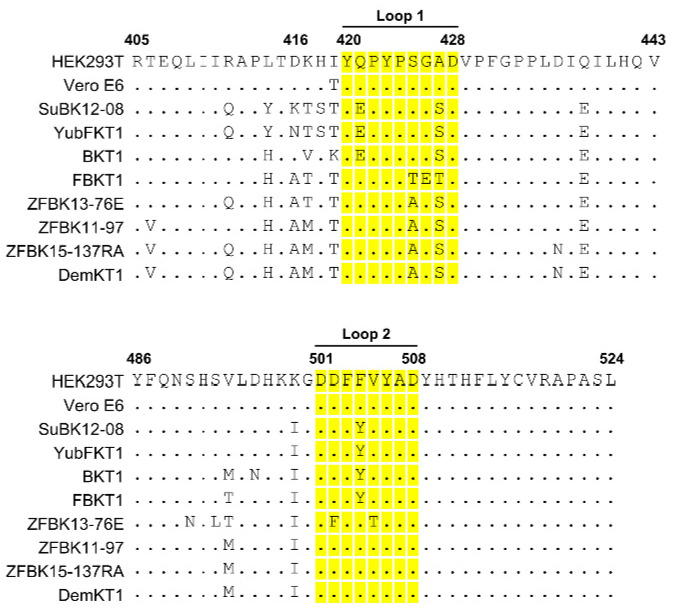
Comparison of amino acid sequences of Niemann–Pick C1 (NPC1) of bat NPC1 orthologues. The deduced amino acid sequences of NPC1-C of SuBK12-08, YubFKT1, BKT1, FBKT1, ZFBK13-76E, ZFBK11-97, ZFBK15-137RA, and DemKT1 are aligned. The amino acid positions of loop 1 and loop 2 are highlighted in yellow.

Since pseudotyped VSVs lean on GP-dependent entry into cells, we assumed that the interaction between GP and its receptor was important for the preferential susceptibility of SuBK12-08 cells to VSV–LLOV. Indeed, the interaction of GP with NPC1, which is believed to be the ubiquitous receptor for filoviruses, including LLOV and newly identified filoviruses [10,11,14], has been demonstrated to control the host specificity of filoviruses [19,20,28,33]. Thus, we compared the amino acid sequences of bat NPC1 domain C (NPC1-C), the region that includes two loop structures which directly interact with the receptor binding domain (RBD) of GP [23,24] (Figure 2). Although there were no unique sequences in the NPC1-C loop regions of SuBK12-08 cells and amino acid residues in the loops of NPC1-C were conserved among three insectivorous bat species, SuBK12-08, YuBFKT1, and BKT1, we found a unique amino acid residue (i.e., lysine [K]) at position 416 in SuBK12-08, near the NPC1-C loop 1, whereas the other two insectivorous bat cell lines had asparagine (N) or aspartic acid (D).

**Figure 3:**
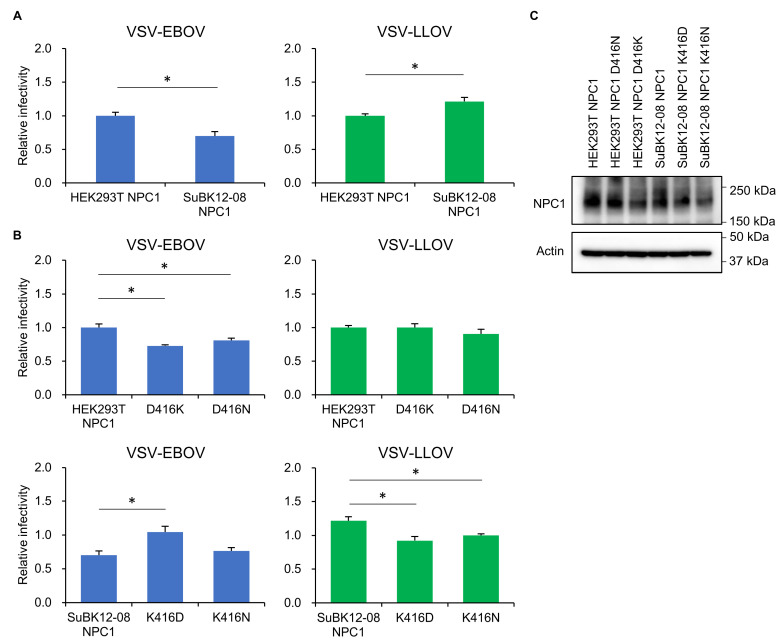
Effect of amino acid substitutions in the NPC1-C on cell susceptibility to pseudotyped VSVs. (**A**,**B**) Vero E6/NPC1-KO cl.19 cells transduced with exogenous NPC1 genes were infected with pseudotyped VSVs. Relative infectivity was determined as described in Section 2. Each experiment was conducted three times, and averages and standard deviations are shown. For comparison of viral infectivity between 2 NPC1-expressing cell lines, Student *t*-tests were performed (**A**). For multiple comparison of infectivities of pseudotyped VSVs in wildtype and mutant NPC1-transduced cell lines, one-way analysis of variance followed by the Dunnett’s test were performed (**B**). Significant differences are shown with asterisks (* *p* < 0.05). (**C**) Expression of wildtype and mutant NPC1. Each cell lysate was subjected to SDS-PAGE followed by Western blotting.

To ascertain whether the difference in the NPC1 sequence between SuBK12-08 and other bat cell lines affected the cell susceptibility to pseudotyped VSVs, we generated Vero E6 cells stably expressing exogenous NPC1 proteins derived from SuBK12-08 and HEK293T, and compared their susceptibilities to pseudotyped VSVs (Figure 3A). Since the full-length sequence of the open reading frame of SuBK12-08 NPC1 was not available, we constructed chimeric NPC1 that had SuBK12-08-derived domain C inserted into HEK293T NPC1 (See Section 2.3). We observed significantly higher infectivity of VSV–LLOV in the cell line expressing SuBK12-08 NPC1 than in the HEK293T NPC1-expressing cell line. In contrast, VSV–EBOV infected the cell line expressing SuBK12-08 NPC1 less efficiently than the HEK293T NPC1 expressing cell line. These results indicated that the amino acid differences between HEK293T and SuBK12-08 NPC1-C were important for the differential cell susceptibility to VSV–LLOV and -EBOV. 

Next, we focused on the amino acid residue at position 416 in NPC1-C as the molecular determinant for the preferential susceptibility of SuBK12-08 cells to VSV–LLOV. We generated Vero E6 cells stably expressing exogenous NPC1 proteins with amino acid substitutions (D416K/D416N and K416D/K416N in HEK293T and SuBK12-08, respectively), and compared the cell susceptibilities to pseudotyped VSVs (Figure 3B). We found that the D416K and D416N mutations reduced the infectivity of VSV–EBOV in the cells expressing human NPC1, whereas these mutations did not affect the infectivity of VSV–LLOV. Interestingly, both K416D and K416N mutations in SuBK12-08 NPC1 significantly decreased the infectivity of VSV–LLOV. Conversely, the K416D mutation significantly increased the infectivity of VSV–EBOV in the cells expressing SuBK12-08 NPC1. The expression levels of exogenous NPC1 molecules were examined by Western blotting and similar band intensities were uniformly detected in each cell line (Figure 3C). These results suggested that the amino acid difference found at position 416 of the NPC1 protein was important for the susceptibility of the *Miniopterus* bat cell line to VSV–LLOV. 

### 3.3. Comparison of Amino Acid Sequences at the NPC1-Binding Interface of Filovirus GP and the Infectivity of VSV Pseudotyped with EBOV, LLOV, and Their Mutant GPs in SuBK12-08 Cells

**Figure 4:**
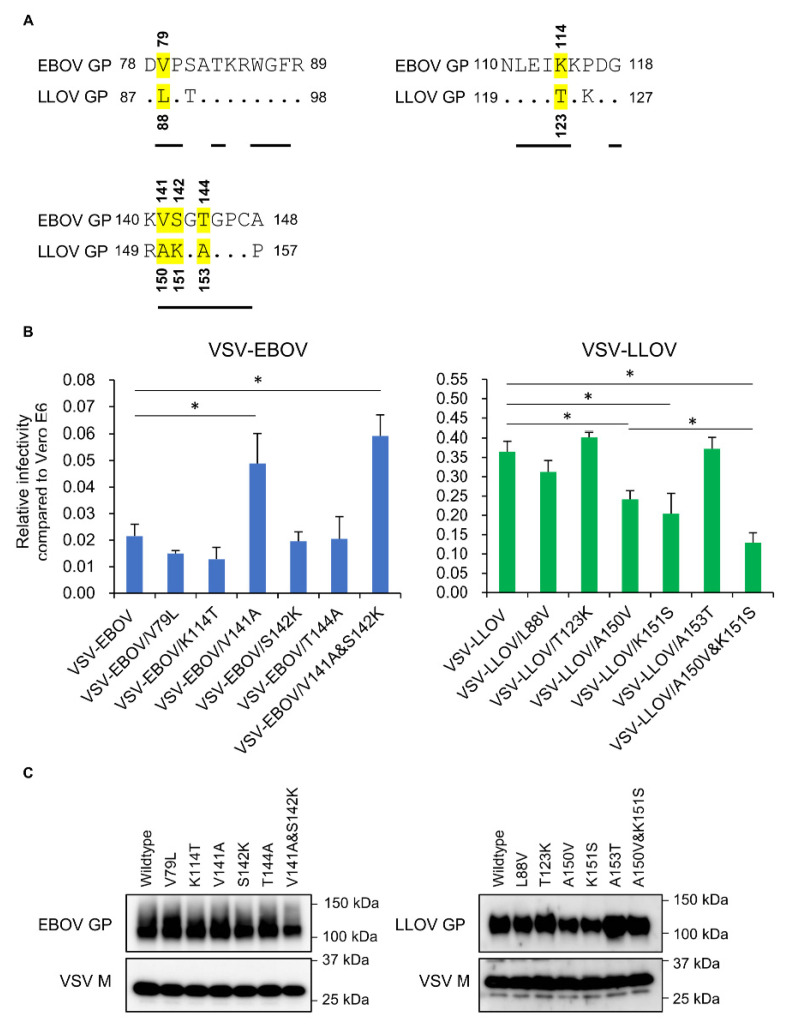
Amino acid differences in the receptor binding domain (RBD) between EBOV and LLOV GPs and effects of amino acid substitutions on the infectivities of pseudotyped VSVs in SuBK12-08 cells. (**A**) The deduced amino acid sequences of EBOV and LLOV GPs are aligned. The amino acid residues predicted to interact directly with the loop structures of NPC1-C, are underlined. The amino acid residues at positions 79, 114, 141, 142, and, 144 (EBOV numbering), which are assumed to interact with loop 1 and loop 2 of human NPC1-C, are highlighted in yellow. (**B**) Vero E6 and SuBK12-08 cells were infected with VSVs pseudotyped with EBOV, LLOV and their mutant GPs. Relative infectivity compared to Vero E6 cells was determined as described in Section 2. Each experiment was conducted three times, and averages and standard deviations are shown. For multiple comparisons of viral infectivities among pseudotyped VSVs, one-way analysis of variance was performed, followed by Dunnett’s test. For comparison of the infectivities of pseudotyped VSVs with single and double mutations, the Student *t*-test was performed. Significant differences are shown with asterisks (* *p* < 0.05). (**C**) The amounts of GPs incorporated into VSV particles. Each virus was subjected to SDS-PAGE followed by Western blotting.

The previously determined cocrystal structure of human NPC1-C and EBOV GP demonstrates that the GP RBD contains key amino acid residues that directly interact with some of the amino acid residues in the loop structures of NPC1-C [24]. Thus, we compared the amino acid sequences around this region in EBOV and LLOV GPs and found five amino acid differences at positions 79, 114, 141, 142, and 144 (EBOV numbering); valine (V), K, V, serine (S), and threonine (T) in EBOV GP and L, T, alanine (A), K, and A in LLOV GP at the corresponding amino acid positions (88, 123, 150, 151, and 153, respectively) (Figure 4A).

To determine the importance of these amino acid residues for the differential infectivity of VSV–LLOV and -EBOV in SuBK12-08 cells, we generated VSVs pseudotyped with GP mutants whose amino acid at position 79, 114, 141, 142, or 144 (EBOV numbering) was swapped between LLOV and EBOV GPs and their infectivities were determined using Vero E6 and SuBK12-08 cells. Though there were no significant differences among VSV–EBOV, -EBOV/V79L, -EBOV/K114T, -EBOV/S142K, and -EBOV/T144A in the relative infectivity in SuBK12-08 cells, VSV–EBOV/V141A showed significantly higher infectivity in SuBK12-08 cells. On the other hand, the infectivities of VSV–LLOV/A150V and –LLOV/K151S were significantly lower than that of VSV–LLOV, whereas there was little difference in infectivity among VSV–LLOV, –LLOV/L88V, –LLOV/T123K, and –LLOV/A153T in SuBK12-08 cells. We also determined the importance of double amino acid substitutions (V141A+S142K in EBOV GP and A150V+K151S in LLOV GP) for the infectivity of pseudotyped VSVs in this *Miniopterus* bat cell line. While there was no significant difference between VSV–EBOV/V141A and -EBOV/V141A+S142K in the infectivity in SuBK12-08 cells, the double amino acid substitution in LLOV GP (A150V and K151S) decreased the infectivity of VSV–LLOV more efficiently than the respective single amino acid substitutions. The GP’s incorporation into VSV particles was confirmed for wildtype and mutant viruses (Figure 4C). These results indicate that the amino acid residue V at position 141 in EBOV GP and A-K at positions 150-151 in LLOV GP played a major role in the differential potential of EBOV and LLOV GP to mediate virus entry into SuBK12-08 cells. 

## 4. Discussion

NPC1 is a ubiquitous receptor for filoviruses, including LLOV, [14,21,22] and has been shown to be required for their cellular entry. It is thus important for their host range restriction and host specificity [19,20,28,33]. In this study, we focused on the interaction between GP and NPC1 to explain the cell tropism of LLOV. We found that the NPC1 protein of SuBK12-08 cells had the potential to mediate LLOV infection more efficiently than those of the others tested (Figure 3A). Although we found no unique amino acid residues in the two GP-interacting loop regions of SuBK12-08 NPC1-C, there was a distinctive amino acid residue (K at position 416) adjacent to the loop 1 region (Figure 2). Therefore, we examined the role of NPC1 K416 in the cell susceptibility to pseudotyped VSVs using a site-directed mutagenesis approach. As expected, substitution of this amino acid position altered the susceptibilities of the cell lines to VSV–LLOV and -EBOV. Although the amino acid residue at position 416 may not be directly involved in the GP-NPC1 interaction, it is possible that this amino acid affects the loop 1 structure, resulting in the difference in the susceptibilities to the viruses among HEK293T, YuBFKT1, and SuBK12-08. It might also be conceivable that K, at this position, increases the interaction between NPC1 and LLOV GP. This possibility must be clarified in future studies.

We found that two amino acid differences between LLOV and EBOV GPs (V and A at position 141, and S and K at position 142: EBOV numbering) were responsible for the differential cell preference of the viruses in SuBK12-08 cells (Figure 4B). It has been shown that V141 and S142 of EBOV GP interact with amino acid residues in loop 1 of NPC1-C [24]. Therefore, these observations support our hypothesis that the mechanism for the preferential tropism of LLOV to *Miniopterus* bat cells may be due to the strengthened interaction between GP and NPC1-C. Furthermore, we also found that the double amino acid substitutions in LLOV GP decreased the infectivity of VSV–LLOV more efficiently than the respective single amino acid substitutions. Since V141 and S142 interact with different amino acid residues on GP (i.e., V141 mainly interacts with Y423 and P424 of NPC1 and S142 mainly interacts with P424, S425, and G426 of NPC1) [24], the amino acid residues at positions 423–426 in NPC1 might be the most important residues for the preferential susceptibility of this bat cell line to VSV–LLOV.

Schreiber’s bent-winged bat (*M. schreibersii*) is an insectivorous bat distributed in southern Palearctic, Ethiopic, Oriental, and Australian regions [34]. Since the discovery of LLOV in Spain [8], anti-LLOV antibodies have been detected in *M. schreibersii* bats there and the LLOV RNA genome has also been detected in the same bat species in Hungary [9,35]. These studies suggest that this bat species may be a preferred host for LLOV. Accordingly, there is a significant difference in the seroprevalence of LLOV infection between bat species (i.e., 36.5% in *M. schreibersii* bats and 0% in *Eptesicus serotine* bats), suggesting that *M. schreibersii* is highly susceptible to LLOV [35] and may be a potential source to spread LLOV to other animal species, including humans and other bats. On the other hand, it still remains to be seen whether *M. schreibersii* acts as the natural host of LLOV since it seems to be pathogenic for this bat species [8,9]. 

Between 11 and 18 species of bat belonging to the genus *Miniopterus* of the family *Miniopteridae* are widely distributed in Europe, Africa, Asia, and Australia [27]. Phylogenetical analyses of cytochrome b genes of *Miniopterus* bats have shown that these bats are divided into European/African species and Australian/Asian species [27], suggesting that the *M. schreibersii* bats in Spain may be genetically more similar to the Zambian *Miniopterus* bat (origin of SuBK12-08) than to *M. fuliginosus* bats (an Asian bat species and the origin of YuBFKT1). Interestingly, these bat species have different amino acid residues in their NPC1 proteins (K416 and N416 in SuBK12-08 and YuBFKT1, respectively). Site-directed mutagenesis revealed that NPC1 with K416 showed an increased ability to mediate VSV–LLOV infection in SuBK12-08, suggesting that the amino acid residues at this position affected the interaction between GP and NPC1. Thus, *Miniopterus* bats in Europe and Africa might have a higher susceptibility to VSV–LLOV than those in Australia and Asia. However, another *Miniopterus* bat species whose NPC1 genome information is currently available (*M. natalensis*; an African bat species) has N416 of NPC1. Therefore, the relationship between the habitat of *Miniopterus* bats and amino acid residues at position 416 of NPC1 needs to be analyzed with a larger number of bat NPC1 sequences.

In this study, we have demonstrated that the interaction between GP and NPC1 is one of the factors controlling the preferential susceptibility of *Miniopterus* bat cells to LLOV. Recently, new filoviruses were discovered in some bat species: Bombali virus in little free-tailed bats (*Chaerephon pumilus*) and Angolan free-tailed bats (*Mops ondylurus*) and Mengla virus in *Rousettus* bats [10,11]. It is important to analyze the host ranges of these newly found filoviruses by focusing on the interaction between NPC1 and GP, since Bombali and Mengla viruses utilize NPC1 as a host cell receptor [10,11]. To understand the whole picture of the filovirus ecology, it is important to determine the tropism and host specificities of newly identified filoviruses as well as previously known human pathogenic ebolaviruses and marburgviruses.

## Figures and Tables

**Table 1:** Origins of cell lines used in this study.

Cell Line	Common Name	Scientific Name ^a^	Organ
Vero E6	African green monkey	*Chlorocebus* sp.	Kidney
SuBK12-08	The long-fingered bat ^b^	*Miniopterus* sp.	Kidney
YubFKT1	Eastern bent-winged bat	*Miniopterus fuliginosus*	Kidney
BKT1	Greater horseshoe bat	*Rhinolophus ferrumequinum*	Kidney
FBKT1	Yaeyama flying fox	*Pteropus dasymallus yayeyamae*	Kidney
ZFBK13-76E	Straw-colored fruit bat	*Eidolon helvum*	Kidney
ZFBK11-97	Peter’s epauletted fruit bat ^c^	*Epomophorus crypturus*	Kidney
ZFBK15-137RA	Egyptian fruit bat	*Rousettus aegyptiacus*	Kidney
DemKT1	Leschenault’s rousettes	*Rousettus leschenaultii*	Kidney

^a^ Scientific names of the species are shown in italics. ^b^ Identified by habitat and nucleotide sequence of the cytochrome *b* gene (99% in BLAST search). ^c^ Identified by habitat and nucleotide sequence of the cytochrome *b* gene (97% in BLAST search). The East African epauletted fruit bat (*Epomophorus minimus*), Ansell’s epauletted fruit bat (*Epomophorus anselli*), Peter’s dwarf epauletted fruit bat (*Micropteropus pusillus*) and Gambian epauletted fruit bat (*Epomophorus gambianus*) are also genetically similar (97%).
